# Isoliquiritigenin inhibits circ0030018 to suppress glioma tumorigenesis via the miR‐1236/HER2 signaling pathway

**DOI:** 10.1002/mco2.282

**Published:** 2023-05-26

**Authors:** Aiqun Liu, Baohong Jiang, Cailu Song, Qizhi Zhong, Yufan Mo, Ruiqin Yang, Ciyu Chen, Cheng Peng, Fu Peng, Hailin Tang

**Affiliations:** ^1^ Department of Neurology School of Clinical Medicine the First Affiliated Hospital of Guangdong Pharmaceutical University Guangzhou China; ^2^ Department of Pharmacy, the First Affiliated Hospital, Hengyang Medical School University of South China Hengyang China; ^3^ State Key Laboratory of Oncology in South China Sun Yat‐sen University Cancer Center Guangzhou China; ^4^ State Key Laboratory of Southwestern Chinese Medicine Resources Chengdu University of Traditional Chinese Medicine Chengdu China; ^5^ West China School of Pharmacy Sichuan University Chengdu China

**Keywords:** isoliquiritigenin, circ0030018, miR‐1236, HER2, glioma

## Abstract

In the central nervous system diseases, glioma is one of the most common malignancies around the world. Despite the recent improvements in therapies for glioma, the prognosis of some high‐risk glioma remains poor. In glioma, isoliquiritigenin (ISL) is reported to have antioxidative and antitumor activities. However, the potential mechanisms between ISL and circle RNAs (circRNAs) in the glioma tumorigenesis process have not yet been reported. Here, we treated glioma cells with ISL, and circRNA expression levels were detected. Circ0030018 was found significantly downregulated by ISL. Therefore, we explored circ0030018 expression profiles and functions in glioma, finding that circ0030018 was evidently overexpressed in glioma cell lines. Colony formation, CCK‐8, and transwell assay made clear that circ0030018 silencing dramatically cut down glioma growth and invasion. Moreover, ROS level was detected to find that circ0030018 silence remarkably enhanced cell oxidative stress in glioma. Mechanism studies were conducted to investigate the underlying basis of circ0030018 function in glioma, unveiling that circ0030018 realized its functions partially through the miR‐1236/HER2 signaling in glioma. In conclusion, our study investigated the roles and mechanisms of the ISL on the circ0030018/miR‐1236/HER2 pathway in glioma tumorigenesis and progression. Circ0030018 could act as the prospective biologic signature or therapeutic target for glioma.

## Graphical abstract

Schematic of this study describing the roles and underlying mechanisms of isoliquiritigenin (ISL) on the circ0030018/miR‐1236/HER2 pathway in glioma progression. Circ0030018 was remarkably overexpressed in glioma and ISL could significantly downregulate circ0030018 expression, leading to dampened glioma growth and metastasis partially through the miR‐1236/HER2 signaling.

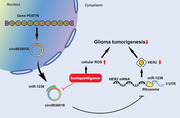

## 1 INTRODUCTION

The incidence of glioma is increasing which leads to an aggressive threat to public health worldwide, accounting for over 70% of malignancies in the central nervous system.^1^ Though the combination of therapies has increased the survival time of some glioma patients, aggressive glioma is still poor. To improve advanced glioma prognosis, the molecular mechanism investigation of this disease is necessary.^2^, ^3^

Studies have indicated that circle RNAs (circRNAs) might play a vital role in nervous system diseases, such as glioma.^4^ For example, circNEIL3 has been reported high expressed in glioma and promoted glioma tumorigenesis and progression.^5^ Additionally, circPTN was significantly upregulated in glioma and could promote glioma growth and stemness via sponging miR‐145‐5p and miR‐330‐5p.^6^ Therefore, it is necessary to further investigate the underlying mechanisms and functions of circRNAs in the malignant process of glioma to develop novel diagnosing biomarkers and treatment targets.

Isoliquiritigenin (2',4',4‐trihydroxychalcone, ISL), is a bioactive compound derived from licorice root. Licorice is commonly known as Glycyrrhiza, which has a wide variety of biological functions and pharmacological effects. And its derivative ISL is involved in the onset and progression of different types of diseases, including cancers. Therefore, ISL could be used as an adjunct treatment for cancers.^7^ ISL has a variety of biological activities including antioxidative as well as antitumor activities.^8^ In glioma, ISL enhanced the temozolomide efficacy by inhibiting the Akt/TGF‐β/VEGF pathway, which might be a potential therapy for glioma.^9^ Besides, ISL inhibited glioma cell growth and induced cell apoptosis by inhibition of the activity of DNA topoisomerase (TOP I).^10^ However, the potential mechanisms between ISL and circRNAs have not been reported in glioma.

Here, we aimed to explore the effect as well as the underlying mechanisms of ISL on circRNAs in glioma and provide a promising antitumor target for glioma treatment. CircRNA expression level detection was performed after cells were treated with ISL. And circ0030018 was found significantly downregulated by ISL. Circ0030018 (chr13:38136718‐38161065) is derived from POSTN, therefore also known as circPOSTN. Circ0030018 has been reported as highly expressed in a variety of cancers, serving as a cancer promotor. Therefore, we investigated circ0030018 expression patterns and functions in glioma cell lines, finding that the levels of circ0030018 were remarkably upregulated in glioma. Following function experiments revealed that inhibition of circ0030018 remarkably weakened cell growth along with invasion capacities, but enhance cell oxidative stress in glioma. Further mechanism experiments suggested that circ0030018 promoted glioma tumorigenesis and progression partially via regulating the miR‐1236/HER2 signaling. Our study investigated the roles as well as the underlying basis of ISL on circ0030018/miR‐1236/HER2 pathway in the glioma tumorigenesis process. Circ0030018 may therefore be used as the prospective biologic signature and therapeutic target in glioma diagnosis and treatment.

## 2 RESULTS

### 2.1 ISL suppresses circ0030018 expression in glioma

**FIGURE 1:**
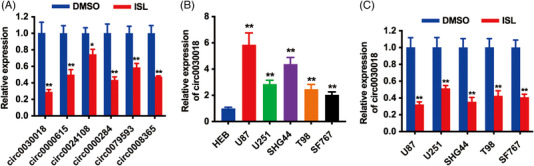
Isoliquiritigenin (ISL) suppresses circ0030018 expression in glioma. (A) The U87 cell line was treated with 5 µM ISL or DMSO as a control for 48 h and then submitted to a quantitative real‐time polymerase chain reaction (qRT‐PCR). Represent circRNA expression levels were shown. (B) The expression levels of circ0030018 in glioma cell lines. (C) Glioma cell lines were treated with 5 µM ISL or DMSO as a control for 48 h and then submitted to qRT‐PCR. Expression levels of circ0030018 were shown. **p < 0.01, *p < 0.05.

For the investigation of ISL effects on circRNAs in glioma, the U87 cell line was treated with 5 µM ISL or DMSO as a control for 48 h. According to a previous study,^10^ the half inhibitory concentration (IC50) of ISL was 6.265 ± 0.89 µM in U87 cells, therefore, we used 5 µM ISL. After that, cells were submitted to a quantitative real‐time polymerase chain reaction (qRT‐PCR) for circRNA expression level detection. As there are a lot of circRNAs that might be regulated by ISL, we chose to verify circRNAs that had been reported to be importantly involved in glioma tumorigenesis and progression in previous studies to explore the functions of ISL. As shown in Figure 1A, ISL suppresses the expression levels of several circRNAs we chose to verify, especially circ0030018. It is reported that circ0030018 takes part in the tumorigenesis process of a variety of cancers. But the effects of ISL on the functions as well as the underlying mechanisms of circ0030018 in glioma remains unreported. Here, qRT‐PCR assays were conducted to assess circ0030018 expression patterns of glioma cell lines. Compared to human normal glial cell line HEB, circ0030018 expressions were elevated in glioma cells, particularly in U87 as well as SHG44 (Figure 1B). Since circ0030018 was upregulated in glioma cell lines, we continued to confirm the function of ISL on circ0030018 expression. The results showed that treatment with ISL could reduce the expression of circ0030018 in a couple of glioma cell lines, particularly in U87 and SHG44 (Figure 1C). All these results revealed that circ0030018 was upregulated in glioma and ISL could suppress the expression of circ0030018 in glioma.

### 2.2 circ0030018 inhibition suppresses glioma proliferation and invasion but enhances cell oxidative stress in vitro

**FIGURE 2:**
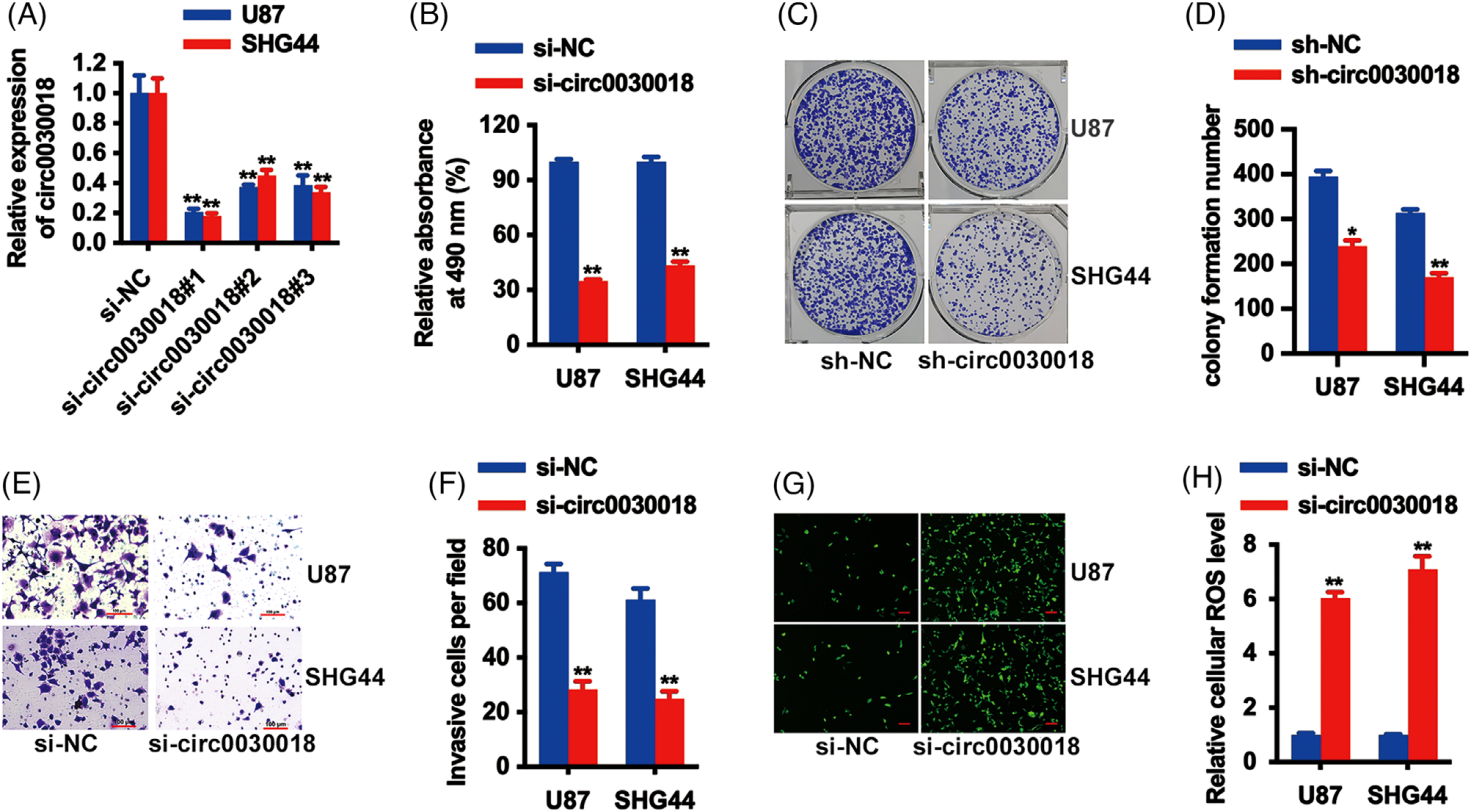
circ0030018 inhibition suppresses glioma proliferation and invasion, but enhances cell oxidative stress in vitro. (A) The knockdown efficacy of si‐RNAs was detected in U87 as well as SHG44 cell lines. (B) CCK‐8 analysis to assess the cell proliferation ability after knockdown of circ0030018 expression. (C) Colony formation assay was used to assess the cell proliferation ability. (D) Statistic graph of colonies. (E) Transwell images of U87 as well as SHG44 cell lines. Scale bar, 100 µm. (F) Number of infiltrative cells was quantified by ImageJ. (G) Representative images of ROS fluorescence by confocal microscopy were shown. Scale bar, 100 µm. (H) Cellular ROS production was detected after circ0030018 inhibition. **p < 0.01, *p < 0.05.

It has been reported that circ0030018 is upregulated in glioma and silencing circ0030018 could hinder glioma progression via regulating the miR‐194‐5p/TRIM44 pathway,^11^ indicating the potential role circ0030018 played in glioma. Due to the overexpression of circ0030018 in glioma, we designed three siRNAs to reduce the level of circ0030018 in U87 as well as SHG44 cell lines to investigate its functions. As Figure 2A showed, circ0030018 levels were remarkably reduced after transfection with si‐circ0030018#1. Therefore, si‐circ0030018#1 was used to construct shRNA for further experiments. Cell proliferation assay conducted using CCK‐8 assays illustrated that circ0030018 inhibition dampened glioma cell proliferation (Figure 2B). Inhibition of circ0030018 evidently suppressed the colony formation potential of glioma cells (Figure 2C,D). Transwell assays presented that circ0030018 inhibition weakened glioma cell invasion (Figure 2E,F). Finally, ROS detection showed that inhibition of circ0030018 increased cellular ROS levels in U87 as well as SHG44 cell lines (Figure 2G,H). All these results revealed that inhibition of circ0030018 could suppress glioma proliferation and invasion, but enhance cell oxidative stress in vitro.

### 2.3 circ0030018 inhibition suppresses glioma proliferation and metastasis in vivo

**FIGURE 3:**
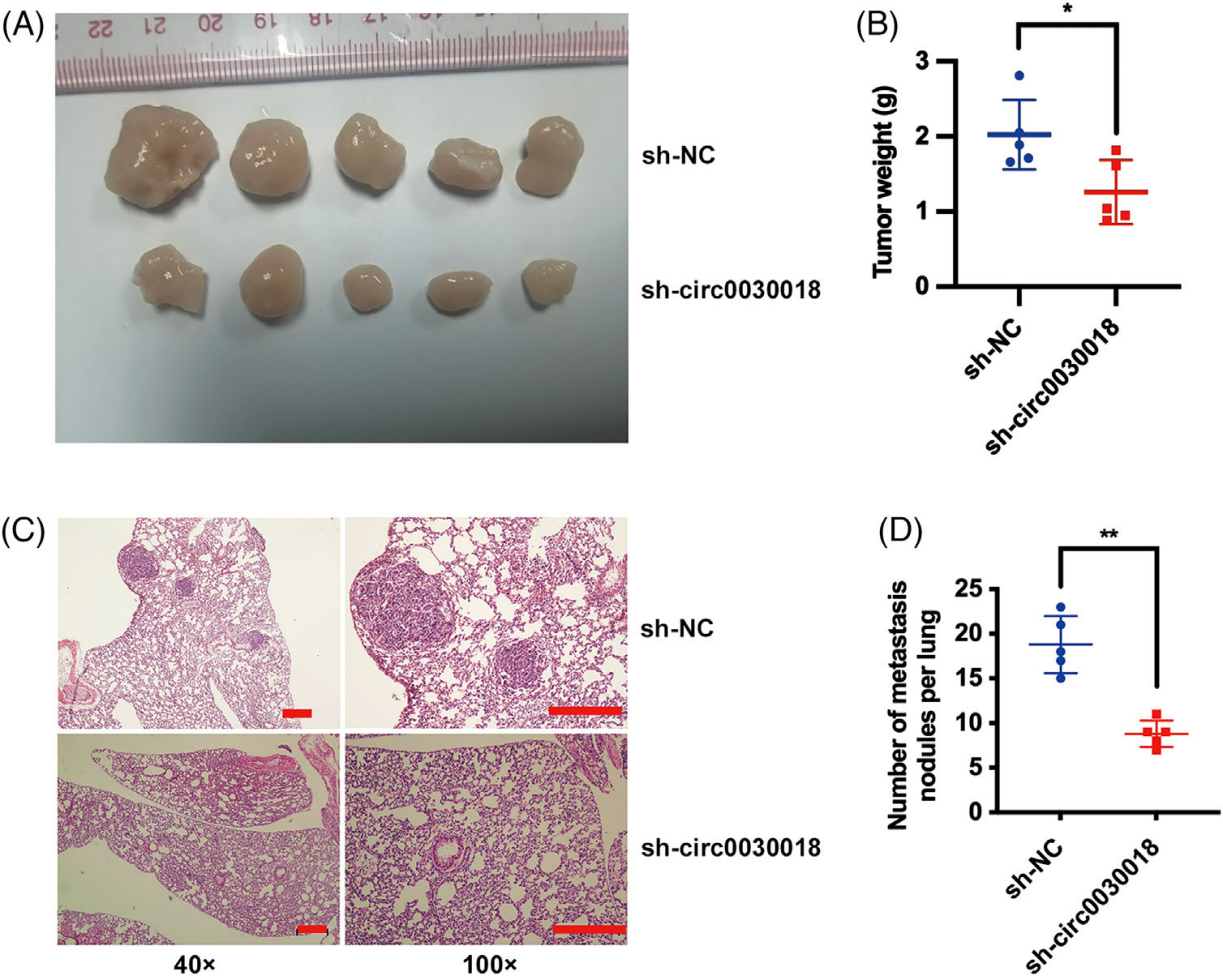
circ0030018 inhibition suppresses glioma proliferation and metastasis in vivo. (A) To explore the functions of circ0030018 in vivo, mouse xenograft models were established. (B) Tumor weights were summarized. (C) Representative images of HE‐stained lung metastatic nodules. Scale bar, 200 µm. (D) Metastatic nodule number was summarized. **p < 0.01.

The in vivo circ0030018 functions in glioma were explored in mice xenograft models. Tumor formation assays manifested that circ0030018 inhibition sharply impaired the growth in vivo (Figure 3A,B). Additionally, lung metastasis models showed that circ0030018 silencing evidently cut down glioma lung metastasis (Figure 3C,D). All these results indicated that inhibition of circ0030018 could suppress glioma growth along with metastasis in vivo.

### 2.4 circ0030018 acts as miR‐1236 sponge in glioma

**FIGURE 4:**
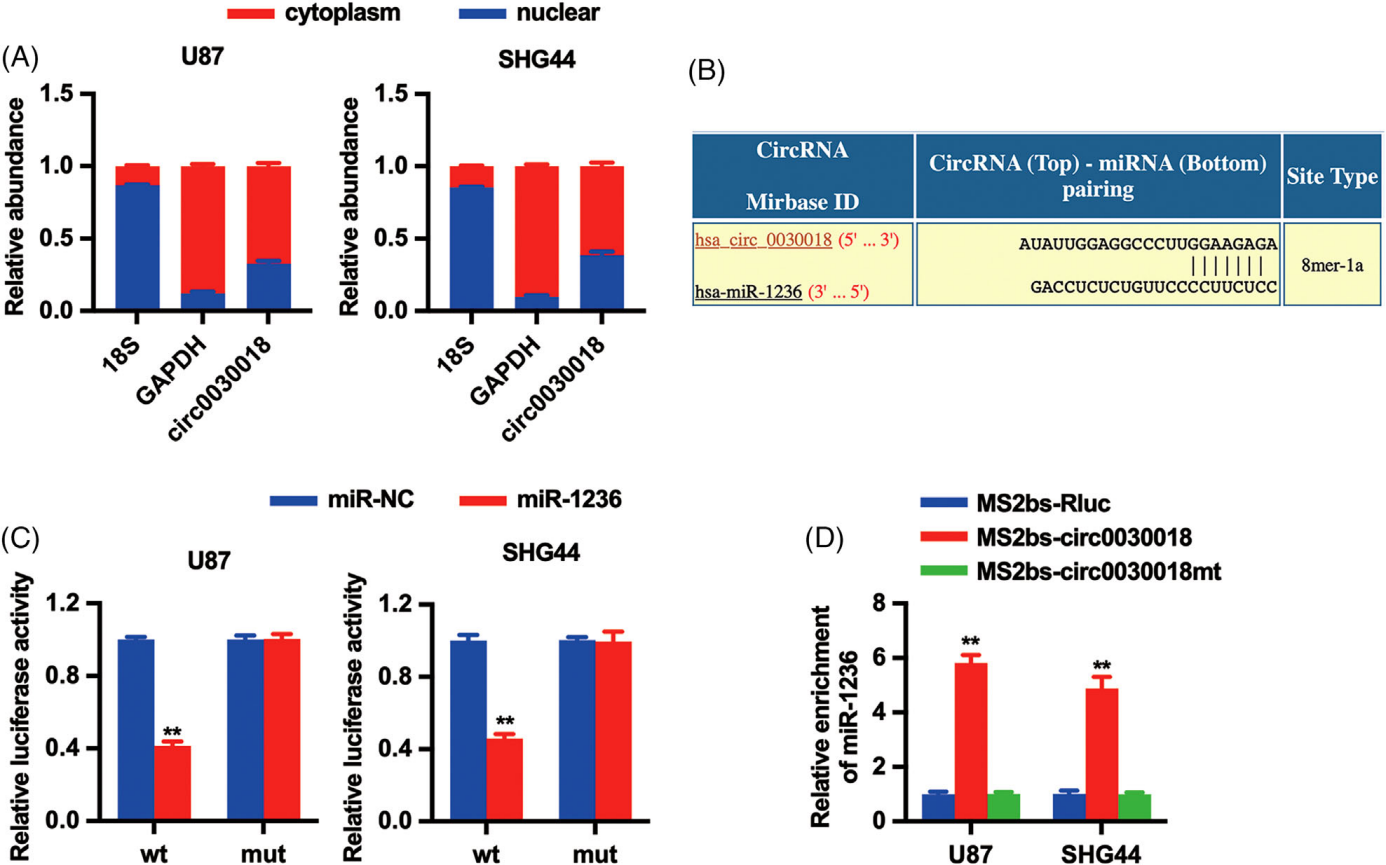
circ0030018 acts as miR‐1236 sponge in glioma. (A) Circ0030018, cytoplasmic control (GAPDH), and nuclear control (18S) expression levels were shown. (B) The predicted miR‐1236 binding sites in the circ0030018 sequence were shown. (C) Luciferase assay was conducted after cell transfection. wt, wild type; mut, mutant. (D) MS2‐based RNA immunoprecipitation (RIP) assay was conducted after cell transfection. **p < 0.01

After functional studies of circ0030018 in glioma, we started to investigate the potential mechanism of circ0030018. Cellular location assay of Circ0038632 found that cell cytoplasm was the predominant localization of circ0030018 (Figure 4A), where the miRNAs predominantly localized, indicating that circ0030018 could realize its functions via acting as a miRNA sponge. Therefore, through bioinformatics analysis, we found potential binding sites for miR‐1236 in circ0030018 (Figure 4B). Thus, a luciferase reporter assay was conducted to confirm the binding. The results revealed that co‐transfection with a wild‐type luciferase reporter and miR‐1236 mimics reduced the luciferase intensity in glioma cells (Figure 4C). Moreover, RNA immunoprecipitation (RIP) assay was performed to further confirm the direct binding and unveiled that miR‐1236 was predominantly enriched in the MS2bs‐circ0030018 group (Figure 4D). All these results indicate that circ0030018 is directly bound to miR‐1236 to serve as a sponge.

### 2.5 circ0030018 regulates HER2 expression via sponging miR‐1236 in glioma

**FIGURE 5:**
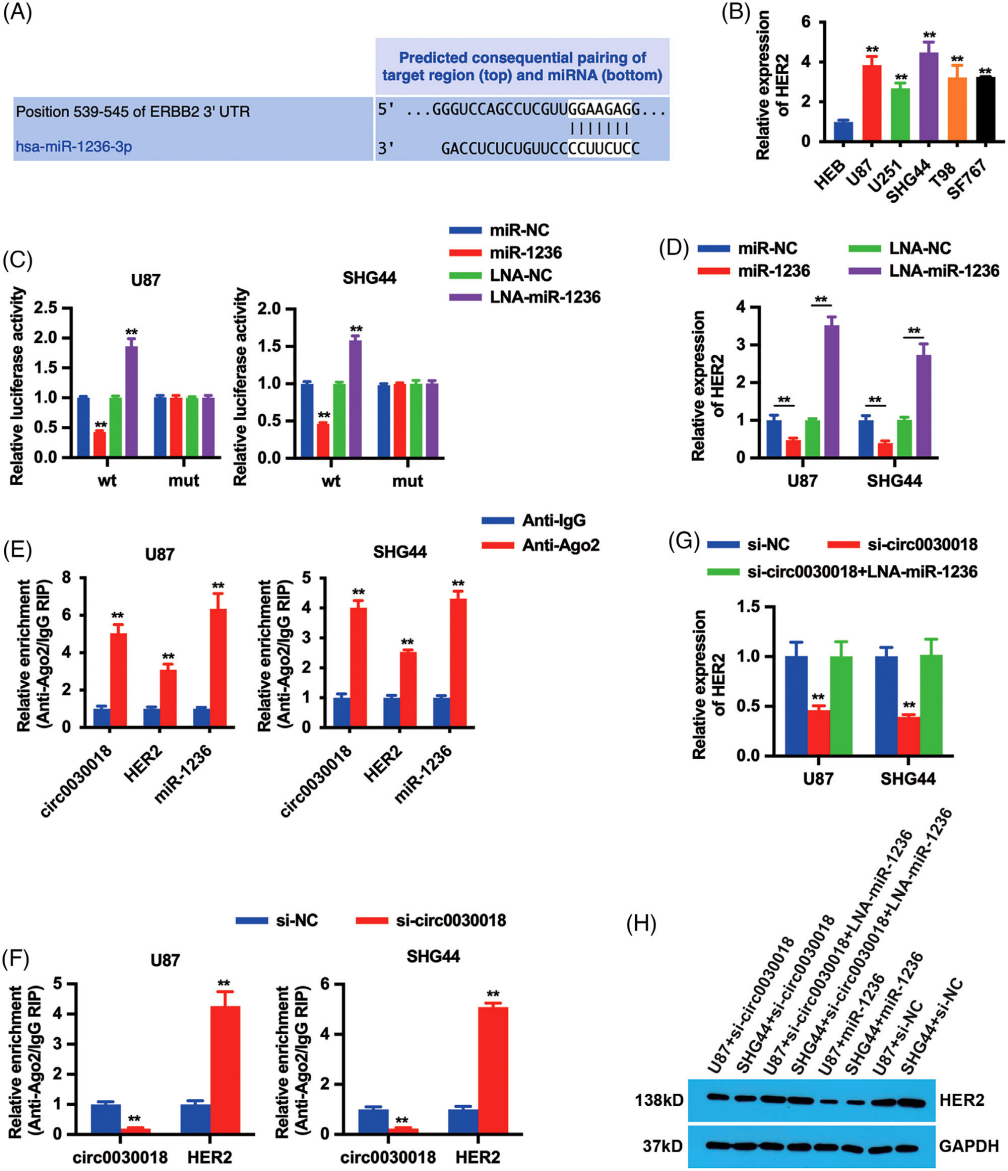
circ0030018 regulates HER2 expression via sponging miR‐1236 in glioma. (A) The predicted miR‐1236 binding sites in HER2 3’UTR. (B) HER2 expression levels in glioma cell lines were detected by quantitative real‐time polymerase chain reaction (qRT‐PCR). (C) Luciferase assay was performed after cell transfection. (D) qRT‐PCR detected HER2 expression after cell transfection. (E) RNA immunoprecipitation (RIP) assay showed the enrichment of circ0030018, HER2, and miR‐1236 to Ago2. (F) RIP assay on Ago2 was performed after cell transfection. (G) qRT‐PCR detected HER2 expression after cell transfection. (H) Western blotting detected HER2 expression after cell transfection. **p < 0.01.

Bioinformatics analysis was performed to explore the potential mechanism and HER2 was sought out to be the potential target gene of miR‐1236 (Figure 5A). HER2 copy number has been reported significantly increased in glioma, which might serve as a prognostic factor and therapeutic target for glioma.^12^, ^13^ Thus, we detected HER2 expression in glioma cell lines and found it increased (Figure 5B). Luciferase reporter assay was conducted and verified the targeting effect of miR‐1236 on HER2. As shown in Figure 5C, co‐transfection with miR‐1236 mimics and wild‐type luciferase reporters reduced the luciferase intensity. Besides, miR‐1236 could decrease HER2 expression levels in glioma cells, while inhibiting miR‐1236 with locked nucleic acid (LNA) enhanced HER2 expressions (Figure 5D), indicating that miR‐1236 could regulate HER2 expression in glioma.

Then, RIP assay on Ago2 was conducted to verify the relationship between circ0030018, miR‐1236, and HER2. As shown in Figure 5E, circ0030018, miR‐1236, and HER2 were all predominantly enriched to Ago2. Besides, circ0030018 inhibition strengthened the enrichment of HER2 to Ago2 (Figure 5F), indicating that circ0030018 could compete with binding miRNAs with HER2. Additionally, circ0030018 inhibition reduced HER2 expression levels, but inhibiting miR‐1236 with LNA could reverse this effect (Figure 5G,H). All these results indicated that the expressions of circ0030018 and HER2 were correlated in glioma, and that circ0030018 could serve as a miR‐1236 sponge to regulate HER2 expression in glioma.

## 3 DISCUSSION

In central nervous system diseases, glioma is one of the most common malignancies around the world, and the incidence is still increasing, leading to an aggressive threat to public health. Despite the recent improvements in therapies for glioma, such as target therapy as well as individualized therapy, the prognosis of some high‐risk gliomas remains poor.^14^ Understanding the mechanism of glioma tumorigenesis is necessary to improve the outcome of aggressive glioma.^15^, ^16^

ISL is a bioactive compound isolated from liquorice and is reported as a potential application in multiple cancers treating glioma included.^7^ The study demonstrated that ISL inhibited glioma stem cell proliferation as well as induced glioma stem cell differentiation via downregulating the Notch1 signaling.^17^ Moreover, ISL inhibited proliferation and induced cell apoptosis by upregulating p21/WAF1 and p27 levels in glioma.^18^ Therefore, ISL could be used as a potential adjunct treatment for glioma. But the effect of ISL on circRNAs and the underlying signaling pathway of ISL anti‐glioma progression are largely unknown. Here, to investigate the effects of ISL on circRNAs in glioma, we treated glioma cells with ISL and found that ISL could suppress the expression levels of several circRNAs, especially circ0030018 (Figure 1).

Increasing studies reported that circRNAs serve important roles in cancers.^19^, ^20^, ^21^ In nervous system diseases, such as glioma, circRNAs are also crucial regulators in tumorigenesis and progression. And circRNAs could be promising diagnostic biomarkers or treating targets for cancer treatments. Circ0030018, also known as circPOSTN, has been reported to be highly expressed in a variety of cancers, promoting the malignant process of cancers. For example, in esophageal carcinoma, circ0030018 was dramatically overexpressed and served as a sponge for miR‐599 to promote cancer progression via regulating the expression of ENAH.^22^ The dysregulation of circ0030018 in glioma development has been reported in a few researches. Circ0030018 was upregulated and its inhibition suppressed glioma proliferation and metastasis in glioma by the miR‐1297/RAB21 axis.^23^ Additionally, circ0030018 is overexpressed in glioma and promotes glioma tumorigenesis through the miR‐185–5p/KIF1B signaling.^24^ Here, we explored circ0030018 expression patterns in glioma cell lines, finding that it was boosted (Figure 1). Moreover, treatment with ISL could reduce the expression of circ0030018, particularly in U87 and SHG44. All the above results revealed that circ0030018 was upregulated in glioma and ISL could suppress the expression of circ0030018 in glioma.

To deeper investigate the potential functions of circ0030018 in glioma, a series of functional experiments such as CCK‐8 assay, colony formation assay, and transwell assay were conducted, revealing that inhibition of circ0030018 significantly suppressed glioma proliferation and invasion (Figure 2). One important characteristic of cancers is cellular energy metabolism reprogramming, leading to enhanced cell growth, invasiveness, and survival of cancer.^25^, ^26^, ^27^ Oxidative stress is involved in various dysfunctions and signaling in cancers.^28^ And ISL is reported to be involved in antioxidation activity in multiple cancers.^29^ Here, ROS detection showed that circ0030018 inhibition enhanced cell oxidative stress in glioma (Figure 2). Moreover, mice xenograft models suggested that circ0030018 inhibition suppressed the growth and metastasis of glioma in vivo (Figure 3). All the above results illustrated that ISL could suppress circ0030018, and inhibition of circ0030018 could suppress glioma growth along with metastasis, but enhance cell oxidative stress in vitro and in vivo, playing an essential role in the glioma tumorigenesis process.

Next, we continued to investigate the mechanism involving ISL/circ0030018 in glioma progress. Increasing studies have demonstrated that circRNAs could regulate cancer progression via sponging miRNAs to regulate the expression of cancer‐related genes, as known as the competitive endogenous RNAs (ceRNA) mechanism. We detected the cellular location of circ0030018 in glioma cell lines and found that circ0030018 predominantly localized in the cell cytoplasm, where the miRNAs predominantly localized, indicating that circ0030018 could realize its functions via acting as a miRNA sponge. Therefore, we decided to explore the ceRNA function of circ0030018 in glioma. Through bioinformatics analysis, we found that circ0030018 might interact with miR‐1236, which is involved in multiple cancer oncogenesis, glioma included. For example, hsa_circ_0074362 sponged miR‐1236‐3p to promote glioma cell proliferation, migration as well as invasion.^30^ Moreover, circ_001350 sponged and reduced the expression of miR‐1236 to exert oncogenic functions in glioma.^31^ But the regulation of circ0030018 on miR‐1236 in glioma remains unknown. Here, the luciferase reporter and RIP assay both revealed that circ0030018 could directly bind to miR‐1236 to sponged miR‐1236 (Figure 4).

In glioma, HER2 amplification was reported to be correlated with poorer survival outcomes and therapy resistance, which could be an independent risk factor.^32^ Moreover, the study shows that R‐LM113, a recombinant HSVs that targets HER2, could lead to effective inhibition of tumor growth in glioma.^33^ Here, bioinformatics analysis showed that HER2 3'UTR had a potential binding site for miR‐1236. Subsequent experiments revealed that miR‐1236 could regulate HER2 expression, indicating that circ0030018 could sponge miR‐1236 to up‐regulate HER2 expression to realize its functions in glioma tumorigenesis. Indeed, RIP assay and HER2 expression detection assays by both qRT‐PCR and western blotting all showed that circ0030018 could sponge miR‐1236 and circ0030018 inhibition reduced HER2 expression in glioma (Figure 5). All the above results indicated that the expressions of circ0030018 and HER2 were correlated in glioma, and that circ0030018 could serve as a miR‐1236 sponge to regulate HER2 expression in glioma.

However, in our study, we failed to verify the expression of circ0030018, miR‐1236, and HER2 in enough patients’ glioma tissue samples. Moreover, the patient clinicopathological parameters and clinical significances of circ0030018, miR‐1236, and HER2 need to be deeper clarified in further study, in order to deeper exploring the potential of circ0030018 as a diagnostic marker and treatment target for glioma.

## 4 CONCLUSIONS

**FIGURE 6:**
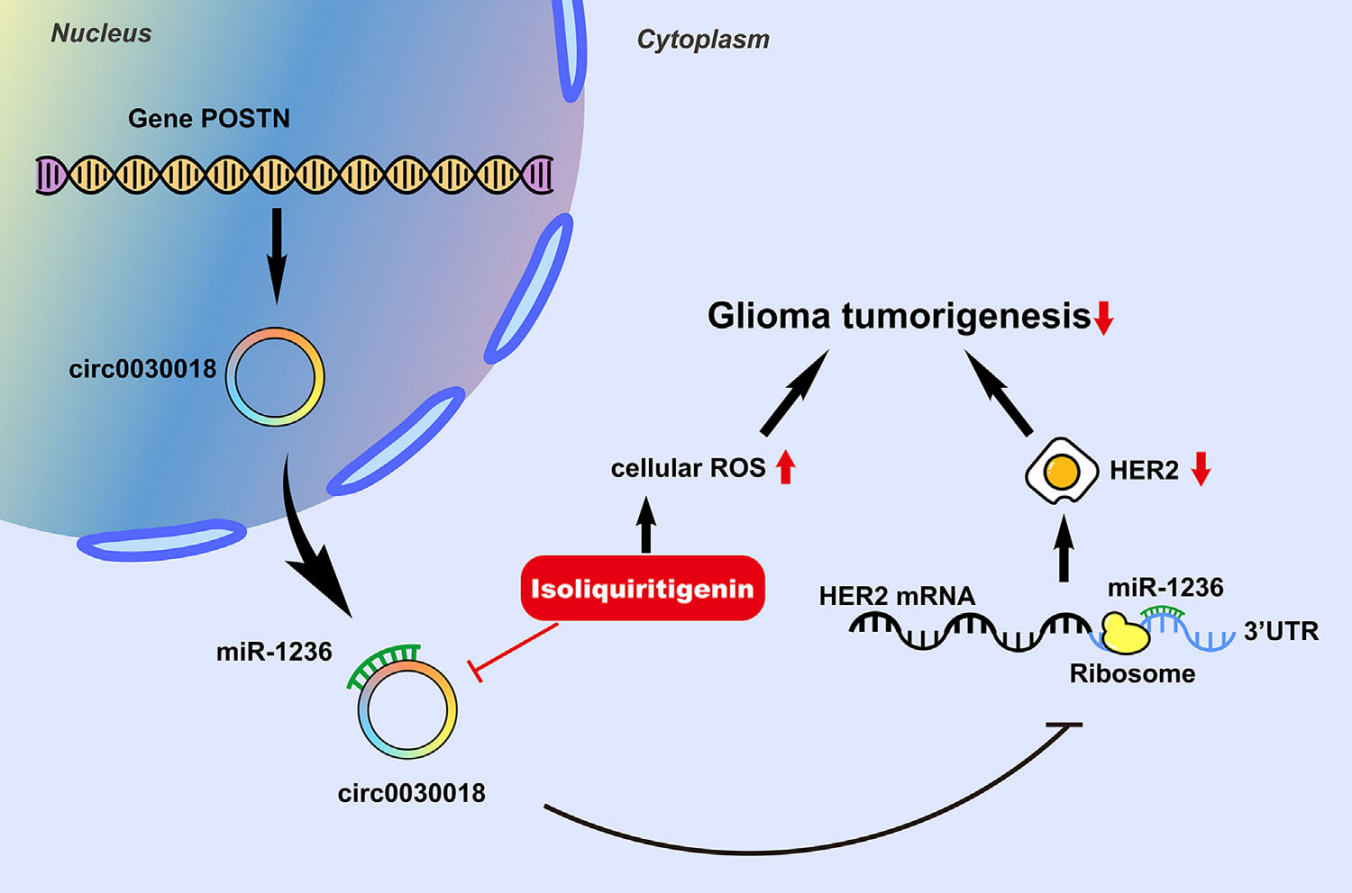
Isoliquiritigenin inhibits glioma tumorigenesis via the circ0030018/miR‐1236/HER2 signaling pathway. Schematic of this study describing the underlying mechanism that isoliquiritigenin inhibits glioma tumorigenesis via the circ0030018/miR‐1236/HER2 signaling pathway.

ISL could suppress circ0030018 expression in glioma. Circ0030018 silencing suppressed glioma proliferation as well as metastasis, but enhanced cell oxidative stress in glioma partially via regulation of the miR‐1236/HER2 signaling (Figure 6). Though the detailed mechanism needs further clarifying, this research disclosed the crucial biological functions of the ISL/circ0030018/miR‐1236/HER2 axis in glioma tumorigenesis progress. Circ0030018 could act as a prospective biologic signature as well as a therapeutic target molecule for glioma.

## 5 MATERIALS AND METHODS

### 5.1 Cell culture

Normal glial cell HEB, as well as glioma cells (U87, U251, SHG44, T98, and SF767) from human beings, were obtained from ATCC (USA) and maintained in DMEM medium (Hyclone, USA) supplemented with 10% FBS (Gibco, USA) at 37°C supplemental with 5% CO_2_ in a humidified atmosphere. Short tandem repeat DNA profiling was adopted for the reauthentication of cells before use.

### 5.2 qRT‐polymerase chain reaction

Total RNA was obtained using Trizol reagent (Invitrogen, USA) and reverse transcription was conducted with PrimeScript RT kit (Applied Biosystems, USA) according to the manufacturer's instructions. mRNA expression detection was conducted with SYBR Premix Ex Taq kit (Takara, China) using the Applied Biosystems 7500 Fast Real‐Time PCR System. The primer sequences used in this study were designed and synthesized by GeneCopoeia (USA) and are provided in Table S1. The relative gene expression levels were calculated by the 2^−ΔΔCT^ formula with GAPDH as the internal control.

### 5.3 Cell transfection

The sequences of the siRNAs used in this study were designed and synthesized by GeneCopoeia and are provided in Table S2. In brief, si‐circ0030018#1, si‐circ0030018#2, si‐circ0030018#3, or si‐NC (control) were transfected to U87 and SHG44 cell lines transiently using Lipofectamine 3000 (Invitrogen) according to the manufacturer's instructions. Cells were collected and submitted to further experiments 48 h later.

### 5.4 shRNA construction and lentiviral infection

shRNA of circ0030018 was designed and synthesized by GeneCopoeia. To package lentivirus expressing sh‐circ0030018, Lenti‐Pac HIV Expression Packaging Kit (GeneCopoeia) was used. After that, the plasmid was transfected to 293 T cells. Then, the culture medium containing lentivirus was collected. Finally, lentivirus was used to infect U87 and SHG44 cell lines with Lipofectamine 3000. And puromycin (2 µg/ml) was used to select stable U87‐sh‐circ0030018 and SGH44‐sh‐circ0030018 cell lines for 2 weeks, which were expanded and submitted to further experiments after that.

### 5.5 CCK‐8 assay

Si‐circ0030018#1 or si‐NC (control) was transfected to U87 and SHG44 cell lines transiently with Lipofectamine 3000 after cells were seeded in 96‐well plates (3 × 10^3^ cells/well each). After being cultured for 48 h, 10 µl CCK‐8 solution (Dojindo, Japan) was added to each well and incubated for 2 h at 37°C. Finally, the optical density value at 490 nM was detected by a microplate reader.

### 5.6 Colony formation assay

U87‐sh‐circ0030018, SGH44‐sh‐circ0030018, and the control cells were seeded in six‐well plates (103 cells/well each) and cultured in a complete medium. 14 days later, the colonies were fixed by 4% paraformaldehyde for 30 min, and stained by 0.1% crystal violet for 30 min at room temperature. The number of colonies (≥50 cells/colony) was counted and averaged in three independent experiments.

### 5.7 Transwell assay

Si‐circ0030018#1 or si‐NC (control) was transfected to U87 and SHG44 cell lines transiently with Lipofectamine 3000. Then the cells were re‐suspended in serum‐free medium and seeded into 24‐well transwell upper chambers (10^4^ cells/well) pre‐coated with Matrigel (BD Bioscience, USA) for invasion assay. Cells in the upper chambers were cultured without FBS, while the lower chambers culture medium was supplemental with 20% FBS solution. After 48 h incubation, the invaded cells were fixed with 4% paraformaldehyde, and crystal violet (0.1%) was used to stain the invaded cells for 20 min. The number of invaded cells was quantified in three randomly selected fields under a light microscope. The original size images of the transwell assay are provided in Figure S1.

### 5.8 Reactive oxygen species detection

Si‐circ0030018#1 or si‐NC (control) was transfected to U87 and SHG44 cell lines transiently with Lipofectamine 3000. Then, cells were seeded on the coverslips in 24‐well plates. After that, cellular reactive oxygen species (ROS) accumulation was detected by a ROS assay kit (Beyotime, China). Cells in serum‐free medium were incubated with 10 µM 2.7‐dichlorofluorescin diacetate for 20 min in the dark at 37°C. Then, cells were washed with PBS three times before examination of fluorescence by confocal microscopy (FV300; Olympus, Japan).^34^ And Image J software was used to quantify average intracellular ROS levels. In brief, the average numbers of cells in the regions of interest (ROI) were counted. And the integrated density value in the ROI was detected and then divided by the cell average number.^35^

### 5.9 Mouse xenograft model

For animal studies, the Sun Yat‐sen University Cancer Center Institute Research Ethics Committee provided the ethical approval (approval number L102032020000D). All the animal studies were performed according to the standard guidelines of the above institution. In brief, male BALB/c nude mice (5 weeks old, weighing 25 ± 4 g) were purchased from the Shanghai Laboratory Animals Center of the Chinese Academy of Sciences. The mice were fed in the standard environment with free access to food or water. 2 × 10^6^ U87‐sh‐circ0030018 cells or U87‐sh‐NC cells were suspended in 0.1 ml physiological saline solution and subcutaneously injected into the dorsal flanks of the male BALB/c nude mice to establish tumors (five mice/group). Finally, after 28 days, the mice were sacrificed and the tumors were excised to measure tumor weight. For lung metastasis model construction, 105 U87‐sh‐circ0030018 cells or U87‐sh‐NC cells were suspended in 0.1 ml physiological saline solution and injected via the male BALB/c nude mice tail vein (five mice/group). After 8 weeks of implantation, the mice were sacrificed and the lungs were excised for following pathology assessment of HE‐staining. Finally, the lung metastatic nodule number was counted and confirmed by microscopy of HE‐stained lung metastatic nodule sections.

### 5.10 Luciferase reporter assay

The fragments containing the predicted binding sites for miR‐1236 in circ0030018 sequence and HER2 3’‐UTR were synthesized and inserted to the pGL3 luciferase vectors (Promega, USA) downstream of the luciferase immediately, served as wild‐type vectors (wt). The mutant vectors with mutations in the predicted binding sites for miR‐1236 were constructed by Fast Site‐Directed Mutagenesis Kit (TIANGEN, China) and served as mutant controls (mut). All the constructs were confirmed before using DNA sequencing. In brief, U87 and SHG44 cell lines were seeded (3 × 10^4^ cells/well each) and then transfected with miR‐1236 mimics or inhibitors and reporting vectors (circ0030018‐wt/mut or HER2 3’‐UTR‐wt/mut) by Lipofectamine 3000. 48 h later, the relative luciferase activities were detected by a dual‐luciferase reporter assay system kit (Promega) using Renilla luciferase activity as a standardized control.

### 5.11 RNA immunoprecipitation

To confirm the binding of circ30018, the MS2‐binding protein (MS2bp)‐MS2‐binding sequences (MS2bs)‐based RIP assays were performed. In brief, U87 and SHG44 cell lines were transfected with MS2bs‐circ0030018, MS2bs‐circ0030018mt (the mutant vector with mutations in the predicted binding sites for miR‐1236), or MS2bs‐Rluc as control vector by Lipofectamine 3000. 48 h later, the RIP assays were conducted with a Magna RIP RNA‐Binding Protein Immunoprecipitation Kit (Millipore, USA). The RNA fraction precipitated by RIP was extracted by Trizol reagent. Then, the level of miR‐1236 was detected by qRT‐PCR.

Additionally, RIP assays for AGO2 were conducted using the above kit. In brief, after transfection for 48 h, RIP assays were conducted with an anti‐Ago2 antibody (Millipore), in which IgG served as a negative control. The RNA fraction precipitated by RIP was extracted by Trizol reagent. Then, the levels of circ0030018, miR‐1236, and HER‐2 were detected by qRT‐PCR.

### 5.12 Western blotting

Total proteins were isolated with radioimmunoprecipitation assay (RIPA) lysis buffer (Sigma, USA) and protease inhibitor PMSF. The concentration of proteins was detected by Pierce BCA Protein assay kit (USA). The protein samples were subjected to 12% SDS‐PAGE for protein separation and then transferred to a PVDF membrane. After that, the membrane was blocked by 5% skim milk powder at room temperature for 1 h. Then, the membrane was incubated with specific primary antibodies against HER2 (1:1000, #ab134182, Abcam, USA) and GAPDH (1:1000, #AF7021, Affinity, USA) at 4°C overnight. Next, the membrane was rinsed by TBST twice, and incubated with horseradish peroxidase (HRP)‐linked secondary antibodies (1:3000, #7074S, CST) at room temperature for 1 h. Finally, the target protein bands were visualized by enhanced chemiluminescence (ECL New England Biolabs, USA). The original data of three independent experiment repeats are provided in Figure S2.

### 5.13 Statistical analysis

Statistical analysis was performed with GraphPad Prism 9.0 software (USA). One‐way analysis of variance along with t‐tests was used in statistical analysis to evaluate differences among different groups. All the data is presented as mean ± SD of three independent experiments. All of the differences were considered statistically significant when *p* < 0.05.

## AUTHOR CONTRIBUTIONS

The authors confirm their contribution to the paper as follows: study conception and design: H. Tang, F. Peng, C. Peng; data collection: C. Chen; analysis and interpretation of results: C. Song, Q. Zhong, Y. Mo, R. Yang; draft manuscript preparation: A. Liu, B. Jiang. All authors have read and approved the final manuscript.

## CONFLICT OF INTEREST STATEMENT

The authors declare no conflict of interest.

## ETHICS STATEMENT

For animal studies, the Sun Yat‐sen University Cancer Center Institute Research Ethics Committee provided the ethical approval (approval number L102032020000D). All the animal studies were performed according to the standard guidelines of the above institution.

## Supporting information

Supporting Information

## Data Availability

All data used and analyzed in this study are available from the corresponding author upon reasonable request. Email should be sent to tanghl@sysucc.org.cn to obtain the shared data.
