# Supplementary material for: Isoliquiritigenin inhibits circ0030018 to suppress glioma tumorigenesis via the miR‐1236/HER2 signaling pathway
Source: MedComm (2020). 2023 May 26;4(3):e282. doi: 10.1002/mco2.282 (PMC10220153; doi:10.1002/mco2.282)
Supplement: Supplementary file 1 — Supporting Information [file MCO2-4-e282-s001.docx]

# Isoliquiritigenin inhibits circ0030018 to suppress glioma tumorigenesis via the miR-1236/HER2 signaling pathway

Running title: Isoliquiritigenin inhibit glioma process

Aiqun Liu^1+^, Baohong Jiang^2+^, Cailu Song^3^, Qizhi Zhong^1^, Yufan Mo^1^, Ruiqin Yang^1^, Ciyu Chen^1^, Cheng Peng^4*^, Fu Peng^5*^, Hailin Tang^3*^

**^1^**Department of Neurology, School of Clinical Medicine, the First Affiliated Hospital of Guangdong Pharmaceutical University, Guangzhou, China

**^2^**Department of Pharmacy, the First Affiliated Hospital, Hengyang Medical School, University of South China, Hengyang, China

**^3^**State Key Laboratory of Oncology in South China, Sun Yat-sen University Cancer Center, Guangzhou, China

**^4^**State Key Laboratory of Southwestern Chinese Medicine Resources, Chengdu University of Traditional Chinese Medicine, Chengdu, China

**^5^** West China School of Pharmacy,Sichuan University, Chengdu, China

**^+^** Aiqun Liu and Baohong Jiang contributed equally to this work.

*****Correspondence: Fu Peng, Email: [pengf@scu.edu.cn](mailto:pengf@scu.edu.cn); Cheng Peng, Email: pengchengchengdu@126.com; Hailin Tang, Email: tanghl@sysucc.org.cn

**
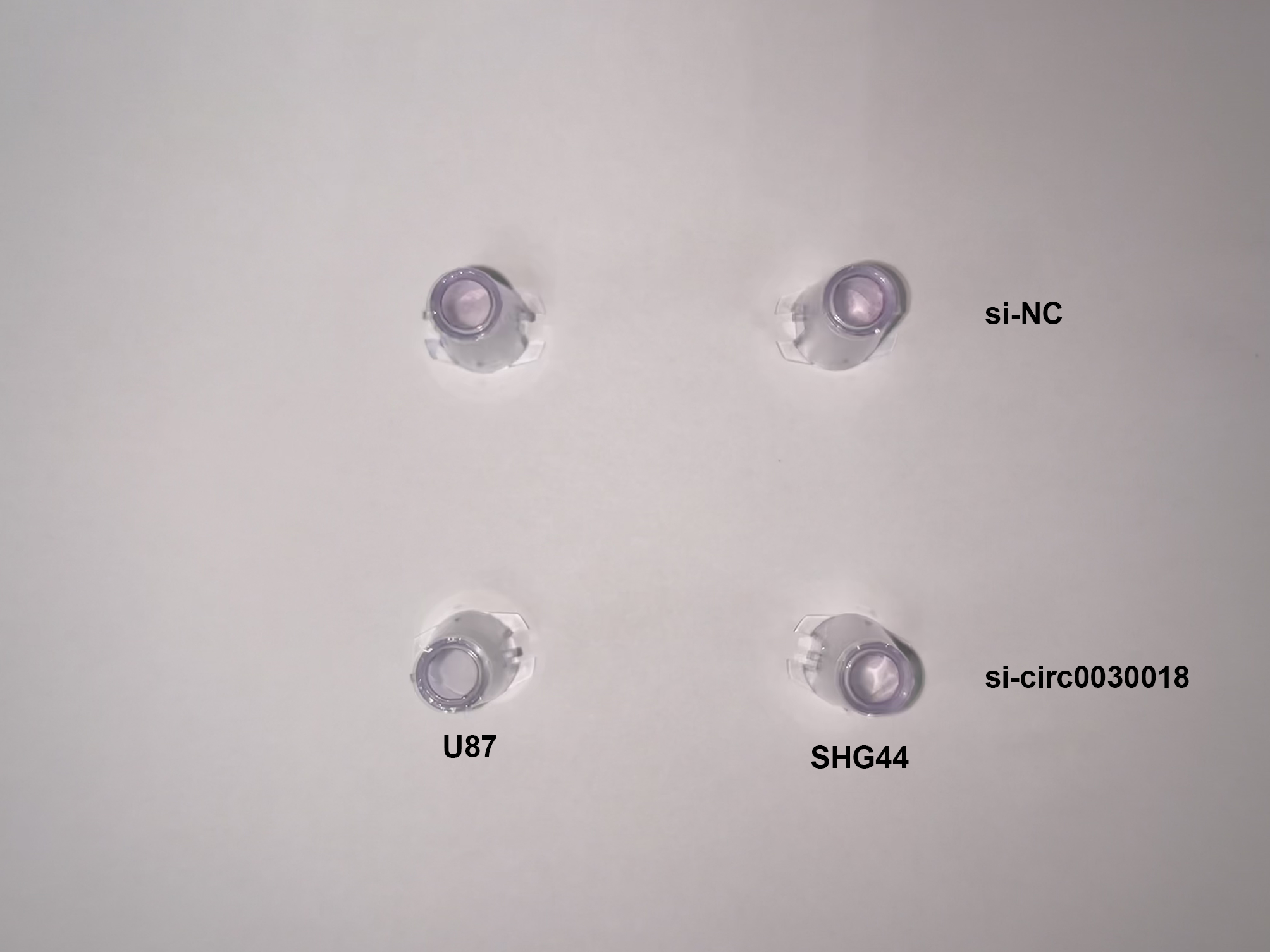
**

**Figure S1. The original size images of transwell assay.**

**
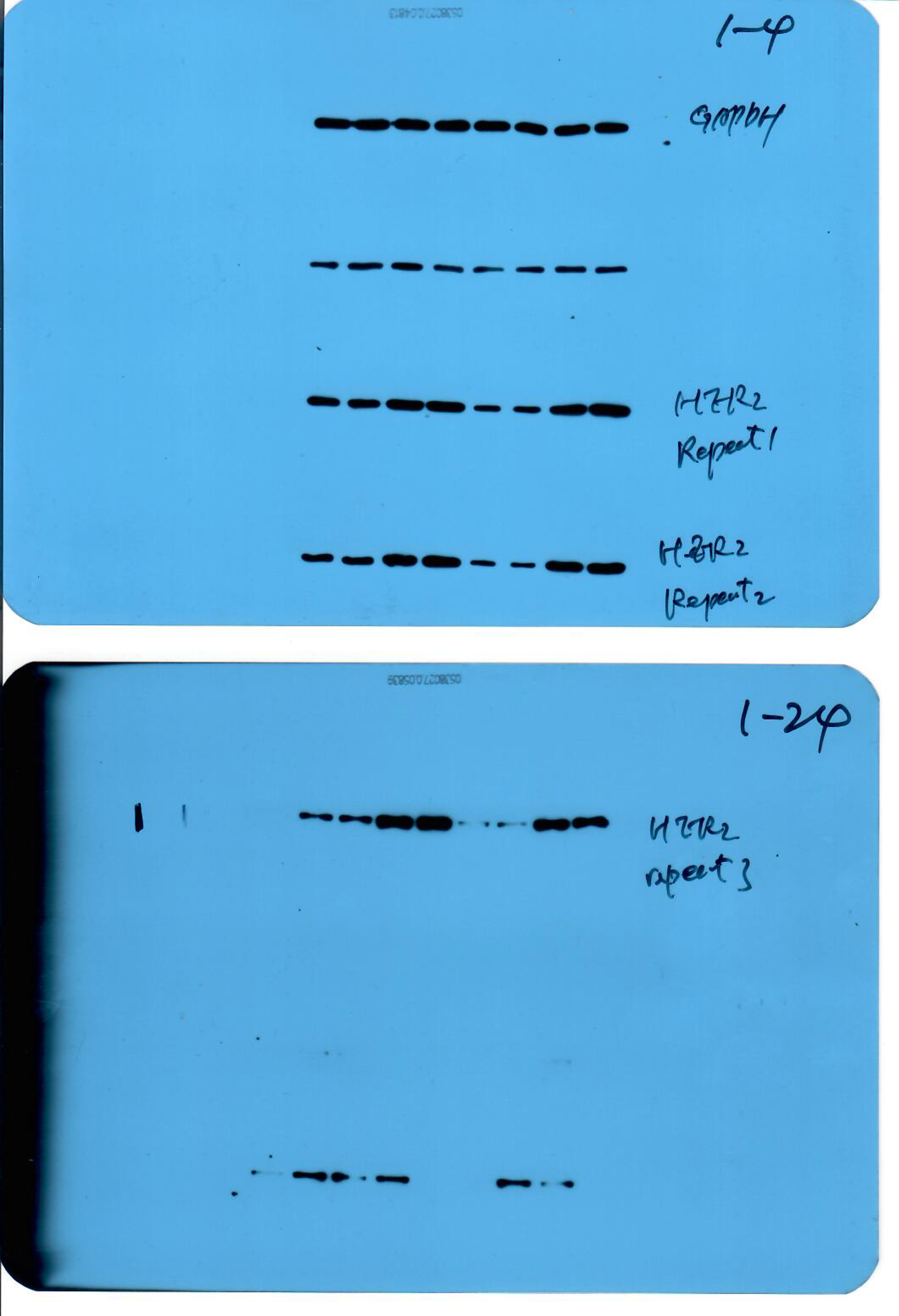
**

**Figure S2. The original data of three independent experiment repeats of western-blot bands.**

**Table S1. The primer sequences for qRT-PCR used in this study**

| ID | Sequence (5’- 3’) |
| --- | --- |
| GAPDH F | TGTTCGTCATGGGTGTGAAC |
| GAPDH R | ATGGCATGGACTGTGGTCAT |
| 18S F | TTAATTCCGATAACGAACGAGA |
| 18S R | CGCTGAGCCAGTCAGTGTAG |
| circ0030018 F | AGTGGTTTGGACTTGGGAAC |
| circ0030018 R | TGGTTCCCATGGATGATTCGAG |
| circ0000615 F | GAATGAGTGTCGCCTGCTAA |
| circ0000615 R | CCCCCAGCTTTCCTATTTTC |
| circ0024108 F | CCTGGATAGGCAAGGGATAA |
| circ0024108 R | GCTTTCTCAATGGCATGGTC |
| circ0000284 F | GGCAGCCTTACAGGGTTAAAG |
| circ0000284 R | GGGTAGACCAAGACTTGTGAGG |
| circ0079593 F | ACTTCAGGGATGCCACCTC |
| circ0079593 R | CTCAGCTTTGGCACATGTCT |
| circ0008365 F | CACAAAGAAACGCACTTTCG |
| circ0008365 R | GGAGATGCCAGTTCATGGTT |
| HER2 F | TGGCCTGTGCCCACTATAAG |
| HER2 R | AGGAGAGGTCAGGTTTCACAC |

**Table S2. The sequences of siRNAs used in this study**

| ID | Sequence |
| --- | --- |
| si-NC | UUCUCCGAACGUGUCACGUTT |
| si-circ0030018 #1 | GAATGAAATTAGTTGTCACTG |
| si-circ0030018 #2 | AAATTAGTTGTCACTGTTAAT |
| si-circ0030018 #3 | AATTAGTTGTCACTGTTAATT |
